# Voice against tobacco: A call for integrated action for effective change

**DOI:** 10.12669/pjms.38.ICON-2022.5788

**Published:** 2022-01

**Authors:** Saima Saeed, Inayat Muhammad Shahroz, Suniya Umar Khan, Alina Ally Agha

**Affiliations:** 1 Dr. Saima Saeed, MRCP (Resp Med) (UK), MSc (UCL) Indus Hospital & Health Network, Indus Hospital & Health Network, Korangi Creek Road, Karachi, Pakistan; 2 Inayat Muhammad Shahroz, MSc Health Policy and Management, Aga Khan University Indus Hospital & Health Network, Indus Hospital & Health Network, Korangi Creek Road, Karachi, Pakistan; 3 Suniya Umar Khan, MSc Mental Health Studies, Kings College London Indus Hospital & Health Network, Indus Hospital & Health Network, Korangi Creek Road, Karachi, Pakistan; 4 Alina Ally Agha, BS Public Relations, University of Texas at Austin Indus Hospital & Health Network, Indus Hospital & Health Network, Korangi Creek Road, Karachi, Pakistan

The use of tobacco amongst the youth of Pakistan is an increasingly concerning issue. Approximately 1,200 children between the ages of 6 and 15 start smoking each day.^1^ As smoking and tobacco use is the single most avoidable cause of death globally, engaging all stakeholders in tobacco control has never been more imperative to improve tobacco control measures and save the future of our youth.

The medical community is well informed of the health hazards of smoking. With lung cancer being an indisputable consequence, tobacco-induced illnesses such as cardio- and cerebrovascular disease, chronic obstructive pulmonary disease and oral cancers are just a few of the other possible outcomes. Particularly for the youth, unique risks include lifelong addiction, sustaining a customer base for the tobacco industry. Pakistan has recently been identified as a business hub by British American Tobacco^2^ and, along with Philip Morris International, actively promotes novel tobacco products in Pakistan. These include electronic devices, ‘heat-not-burn’ tobacco and nicotine lozenges such as Velo, making tobacco use modern and trendy. To address the tobacco industry’s innovation and targeted advertising, integrated methods must be leveraged and awareness created around risk perceptions of young people to subsequently fuel tobacco control efforts.

Interventions by health workers may be highly effective in individual smoking cessation efforts, with even a 3-minute counselling session resulting in benefits. Behavioural change and pharmacological interventions are also available. Beyond this, health professionals can involve themselves by building relationships with stakeholders and policy makers, speaking up or writing about the issue at hand. In Pakistan, however, where tobacco has become a cultural norm, physicians do not fully exploit this power to enable actionable change in tobacco uptake. Realizing their role as important societal leaders who can ‘denormalise’ tobacco use, particularly amongst youth, is critical.

Pakistan is a signatory to the World Health Organization (WHO) Framework Convention on Tobacco Control (FCTC), supporting the implementation of comprehensive tobacco control programs through MPOWER. MPOWER employs the following strategies: Monitor tobacco use and interventions, protect people from tobacco smoke, offer help to quit tobacco use, warn about the dangers of tobacco, enforce bans on tobacco advertising, promotion and sponsorship, and Raise tobacco taxes and develop sustainable alternatives to tobacco growing. Yet the complex tobacco tax structure in Pakistan, providing economic favor to the tobacco industry, appears counterintuitive to this.

A 10% increase in prices would reduce tobacco use in teenagers by 18%.^3^ Therefore, because the youth is more sensitive to the price of goods, making tobacco products less affordable is likely to have a direct impact on their consumption. Disconnected tobacco control advocacy and intense lobbying by the tobacco industry leads to an impasse and so, groups must band together in an effort to raise awareness about required policy changes. A de facto coalition must include the medical community, civil society organizations, youth groups, academic institutions, public health professionals and policy makers. Only then can individual efforts be unified, strengthened and channeled for effect.

Indus Hospital & Health Network has used the MPOWER framework to create ‘Voices Against Tobacco’ (VAT). This is a community-centric initiative aimed at supporting positive policy change and empowering Pakistan’s youth to take ownership of their health and future. VAT is a direct response to the need for a tobacco control coalition in Pakistan and the objective of this is simple - to provide a common platform for anti-tobacco advocates to amalgamate and support initiatives against tobacco use.

VAT aims to invest in Pakistan’s youth by conducting capacity building exercises in school-based tobacco control training. VAT champions will then advocate for enhanced tobacco control measures amongst their communities and collect narratives on tobacco use, advertising and pricing. This will support galvanization of a strong group amongst the youth and project their advocacy efforts through multiple stakeholder engagement sessions and social media campaigns. Community outreach, along with a concurrent media campaign, will support national policy objectives including tobacco taxation, novel products regulation and sustainability of tobacco control through engagement with policy makers.

Protecting the health, well-being and livelihood of Pakistani youth is of utmost importance, for their future and the future of the country. By synergizing multiple groups of value to tobacco control efforts, we can push for effective policy change and facilitate meaningful change in the current state of tobacco in Pakistan.
